# Massive Tibial Bone Regeneration with Autologous Peripheral Blood Stem Cells using Ilizarov Bone Transport: A Case Report

**DOI:** 10.5704/MOJ.2011.026

**Published:** 2020-11

**Authors:** KY Saw, R Gill, TC Low

**Affiliations:** Department of Orthopaedic Surgery, Kuala Lumpur Sports Medicine Centre, Kuala Lumpur, Malaysia

**Keywords:** massive bone loss, bone regeneration, Bone Healing index, peripheral blood stem cells, Ilizarov

## Abstract

This is a case report of a Gustilo-Anderson Type IIIB comminuted open right tibial fracture with massive bone loss, complicated by methicillin-resistant Staphylococus aureus (MRSA) infection. Non-viable and contaminated bony fragments were removed and infected bone resected. Soft tissue coverage and antibiotics were effective against the MRSA infection. A unifocal bone transport with the Ilizarov method regenerated 13cm of the missing tibia. Autologous peripheral blood stem cells (PBSC) injections into the osteogenesis site boosted bone regeneration and consolidation with a shortened Bone Healing index (BHI) of 23 days/cm.

## Introduction

Managing massive bone loss following traumatic open fracture is challenging as normal healing is compromised by poor vascularity. Recent studies support the use of stem cells for musculoskeletal applications and improving the reparative process^1-3^.

We present our experience with Ilizarov distraction osteogenesis (DO) boosted by injections of autologous peripheral blood stem cells (PBSC) to enhance bone regeneration and consolidation, thus shortening the Bone Healing index (BHI). The BHI relates to “the length of time a patient undergoes treatment with an external fixator to the measured length of bone gained”^4^.

## Case Report

**Fig. 1:**
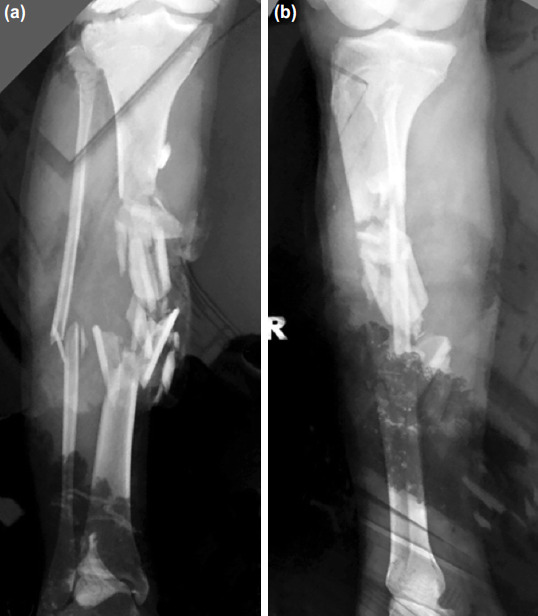
(a) Radiographs with anteroposterior (b) and lateral views of the right tibia showing severe comminuted fracture of the middle third of the tibia.

A 16-year-old Grand Prix motorcycle rider collided with another rider at a speed of over 280 km/h, just before the chequered flag. As he drifted across the racetrack, an oncoming bike ran over the middle third of his right tibia. He sustained a Gustilo-Anderson Type IIIB comminuted open tibial fracture with massive bone loss (Fig. 1). The comminuted bony fragments were removed, wound debridement performed, and an external fixator applied. There were no other life-threatening injuries apart from ipsilateral anterior cruciate ligament and medial collateral ligament ruptures with neurapraxia of the common peroneal and tibial nerves.

Transferred to our care 48 hours after the original injury, he underwent further wound debridement with meshed split skin graft applied over the exposed soft tissues. An Ilizarov frame was applied for bifocal bone transport one week later. Proximal corticotomy was performed below the tibial tuberosity and distal corticotomy was performed above the ankle joint. An ankle diastasis screw was inserted, which was subsequently removed at six weeks.

One week after the Ilizarov procedure, PBSC were harvested. The details of the harvesting procedure and cell preparation are outlined in our previous publication^1^. One day after the apheresis process, 8ml of PBSC were injected into each of the proximal and distal corticotomy sites.

Four weeks after admission, the wound swabs grew methicillin-resistant Staphylococus aureus (MRSA) which was sensitive to linezolid. Unfortunately, the MRSA infection resulted in proximal wound breakdown and exposure of the distal end of the proximal corticotomy site. This required shortening and excision of the exposed bone to enable soft tissue coverage. At the same time, the proximal bone lengthening corticotomy site was reversed to allow re-approximation of the corticotomy bone ends. We then decided to only perform unifocal bone transport from the distal corticotomy site.

DO commenced the day after the distal corticotomy without the usual latent period (initially at a rate of 1mm lengthening once per day, and then varied between 1mm and 2mm during the lengthening process) governed by radiographic evidence of osteogenesis. A two-monthly injection regime of 12ml of PBSC into the distal corticotomy site and into the middle of the regenerating bone was instituted.

**Fig. 2:**
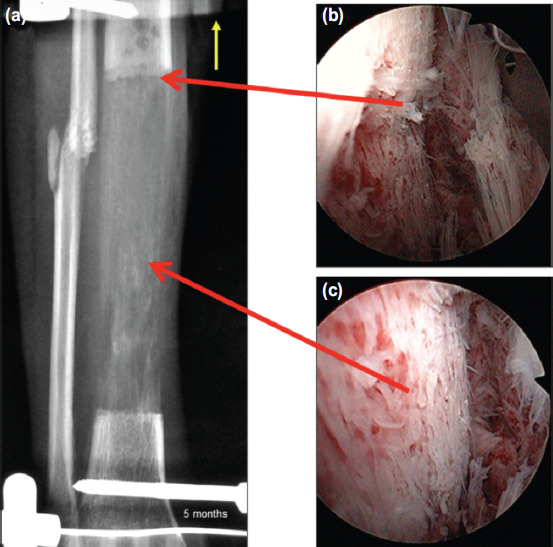
(a) Bone transport at five months showing distraction osteogenesis over the distal half of the right tibia, lower horizontal Schanz screw inserted just above the ankle joint with yellow arrow indicating the direction of bone transport from the ankle corticotomy site; (b) Endoscopic view from the posterior aspect of the tibia following soft tissue release with red arrow at the junction of the corticotomy and the edge of bone regeneration; and (c) Endoscopic view from the posterior aspect of the regenerating bone with red arrow at the middle section of the distraction osteogenesis showing spongy nature of the regenerated bone.

Two months into the DO process, progressive equinovarus deformity of the foot and ankle was noted and the soft tissues adherent to the posterior aspect of the transported tibia segment were released endoscopically (Fig. 2). This procedure was repeated three months later.

**Fig. 3:**
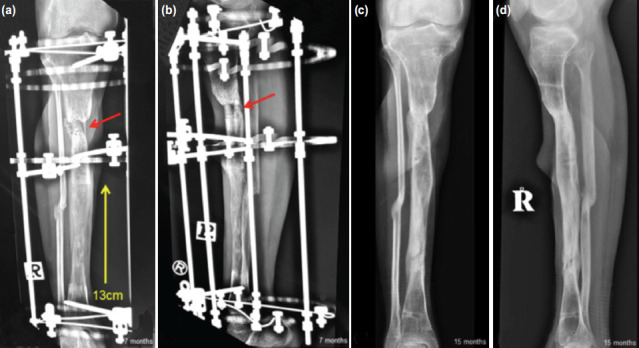
(a) Anteroposterior and (b) lateral radiographs of the right tibia at seven months following bone transport with the Ilizarov method. Red arrows showing multiple drilling over the docking site performed two months previously with injections of peripheral blood stem cells to enhance callus formation. Yellow arrow indicating the direction of bone transport from the ankle corticotomy site, showing 13cm of bone regeneration over the distal half of the tibia. Similar views (c) and (d) at 15 months, showing bone healing at the docking site and further consolidation of the regenerated bone.

DO was continued for five months with eventual docking of the bone ends. During the docking process, debridement of the surrounding soft tissues was made, followed by multiple drilling into the bone ends and injection of 8ml of PBSC into the docking site (Fig. 3a, b). A distally based fasciocutaneous flap was also fashioned over the anterior aspect of the proximal tibia to improve soft tissue coverage. The Ilizarov frame was removed ten months after regenerating 13cm of bone over the distal half of the tibia, giving a BHI of 23 days/cm (Fig. 3). A protective thermoplastic tibial brace was tailored for the healing tibia following the removal of the frame.

Throughout the process of bone regeneration, the pin sites were cleaned daily and the patient was prescribed calcium and vitamin B complex supplements. A twice-daily inpatient physiotherapy regime was implemented with progressive weight-bearing exercises, ultrasound and soft tissue electrical stimulation, and strengthening and mobilisation of the knee and ankle joints, together with general body conditioning.

The neurapraxia of the common peroneal and tibial nerves gradually improved and nerve conduction studies showed almost normal results at 12 months. The equinovarus deformity of the foot and ankle was corrected at this stage by lengthening the Achilles tendon, releasing the spring ligament and subtalar joint and re-routing the posterior tibial tendon to the anterolateral aspect of the foot, attaching it to the peroneus brevis tendon. This improved the patient’s gait and allowed a progression to full weight-bearing.

The patient went back to professional riding 15 months after the original injury, with no evidence of bone infection, while awaiting an appropriate time to undergo right knee reconstructive surgery.

## Discussion

It is clear that PBSC played an important role in this enhanced DO. We believe that 13cm of tibial bone regeneration over a period of ten months from a unifocal lengthening with a seemingly shortened BHI of 23 days/cm has not been previously reported.

Alkenani *et al* published a 14.5cm tibial bone regeneration from a unifocal tibial lengthening and reported a BHI of 52 days/cm^5^. Saw *et al* previously reported a BHI averaging 36 days/cm in a case series^4^.

We have previously published on the application of PBSC for bone, cartilage and soft tissue regeneration, including its safety and efficacy, in a randomised controlled trial^1,2^. PBSC comprise haematopoietic stem cells, mesenchymal stem cells (MSC) and growth factors and are able to differentiate into the mesenchymal lineage, including bone and cartilage. We theorise that PBSC perform two functions during osteogenesis: (1) the integration and differentiation towards the osteogenic lineage, and (2) the activation of PBSC to release trophic factors into the local environment, augmenting endogenous MSC recruitment from the bone marrow. This results in enhanced osteogenesis and consolidation, hence the shortened BHI in this case when compared to the results of other published studies^4,5^.

The take-home message from this report is that the potential of stem cells is enormous and it is up to the treating surgeon to look at evidence-based medicine to push the frontiers of medicine for the benefit of our patients.

## References

[1] Saw KY, Anz A, Jee CSY, Merican S, Ng CSR, Roohi SA (2013). Articular cartilage regeneration with autologous peripheral blood stem cells versus hyaluronic acid: A randomized controlled trial. Arthroscopy..

[2] Saw KY, Jee CSY. From (2013). ‘blade runner’ to ‘stem-cell player’ and beyond. Bone Joint 360..

[3] Yang Y, Lin S, Wang B, Gu W, Li G (2017). Stem cell therapy for enhancement of bone consolidation in distraction osteogenesis. Bone Jt Res..

[4] Saw A, Manimaran S, Faizal S, Bulgiba AM (2008). Use of radiographic densitometry to predict the bone healing index in distraction osteogenesis. Malays Orthop J..

[5] Alkenani NS, Alosfoor MA, Al-Araifi AK, Alnuaim HA (2016). Ilizarov bone transport after massive tibial trauma: case report. Int J Surg Case Rep..

